# A glassware-free combinatorial synthesis of green quantum dots using bubble wrap†

**DOI:** 10.1039/c9ra02018g

**Published:** 2019-05-29

**Authors:** P. Bergstrom Mann, K. Afzal, N. J. Long, M. Thanou, M. Green

**Affiliations:** Department of Physics, King's College London The Strand London WC2R 2LS UK mark.a.green@kcl.ac.uk; Department of Chemistry, Imperial College London, Molecular Sciences Research Hub White City Campus, 80 Wood Lane London W12 0BZ UK; Cancer and Pharmaceutical Sciences, King's College London Franklin Wilkins Building London SE1 9NH UK

## Abstract

Here, we describe the use of commercially-available bubble wrap as the basis for the simple, cheap combinatorial exploration of the synthesis of brightly emitting core/shell quantum dots.

## Abstract 2

In this communication, we highlight the use of bubble wrap in the simple parallel synthesis of CuInS_2_-based quantum dots with different optical properties, based on varying precursors concentrations.

## Graphical abstract

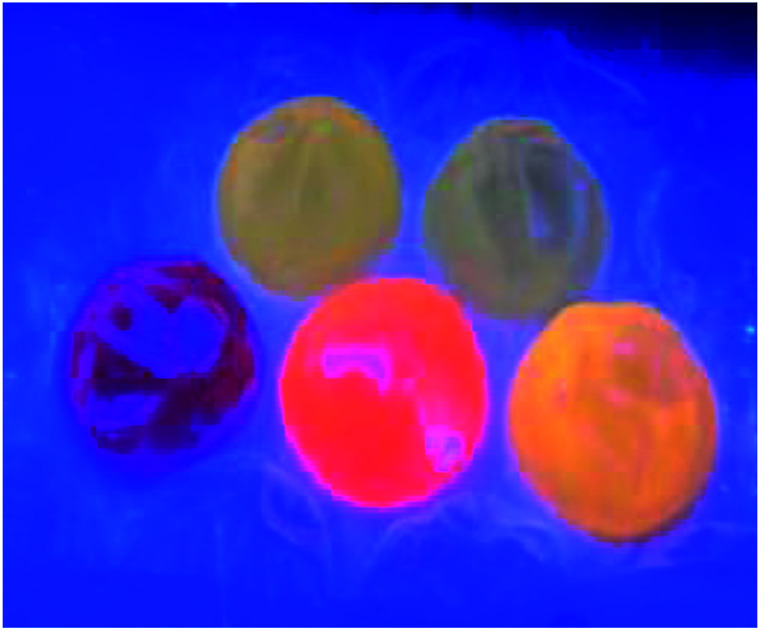

New chemical compounds are discovered at an astonishing rate; in 2015, the Chemical Abstract Service recorded its 100 millionth compound. Despite this, the actual hardware associated with synthetic chemistry has evolved at a much slower rate. Most chemical glassware is based on borosilicate glass (developed in 1915), with quartz, actinic and PTFE-coated glass used for analysis and storage.^1^ There have been few developments in glassware until recently, when the Cronin group reported 3D-printed acetoxy silicone reaction-ware that could be used for cluster synthesis, with the geometry of the reaction vessel dictating the actual reaction product.^2^ Further advances include the use of 3D-printed polypropylene modules that were combined and used to synthesise a simple drug.^3^

One of the key developments in how reactions are carried out was the emergence of combinatorial chemistry and parallel synthesis, providing the ability to implement numerous chemical reactions simultaneously. When applied to material science, the seminal studies in combinatorial materials chemistry explored the preparation of superconducting materials using vapour deposition masks, allowing a library density of 10 000 samples per square inch.^4^ The emergence of materials chemistry has expanded beyond simple deposition techniques, and there exists a requirement for parallel solution chemistries to be developed. The use of combinatorial chemistry in material and nanoparticle science is well-documented, with luminescent materials (in which we are primarily interested) representing only a small fraction of what can be explored.^5^ Undertaking such work can be, however, expensive, requiring a large initial outlay for robotics kit for example, which makes such chemistry prohibitive for some developing laboratories. Also, not all combinatorial experiments need a sample density in the thousands; for some, tens of outputs are sufficient to arrive at a suitable positive outcome. Similarly, solution-based material science cannot rely on vapour deposition techniques.

An important nanomaterial developed during the last three decades are the quantum dots. A combinatorial approach to the synthesis of CdSe quantum dots has been reported although this method utilised several microreactors and is beyond most synthetic laboratories.^6^ Likewise, a high-throughput robotic system has been used to explore the reproducible synthesis of various luminescent nanoparticles, giving unrivalled control and optimisation of the reactions; again, the instruments used in such a study are equally unique.^7^

Inspired by a report from the Whiteside group which outlined the use of bubble wrap to store liquids, culture bacteria and take optical and electrochemical measurements, we describe the use of commercial bubble wrap as a simple reaction vessel and demonstrate multiple simultaneous reactions as a proof-of-principle for its use in basic combinatorial-style chemistry.^8^ Bubble wrap, as highlighted by Bwambok *et al.*, has a variety of positive attributes that makes it ideal for simple aqueous-based chemistry. Of particular note are the low cost, ease of manipulation and disposal and the density of bubbles (library density) of up to 5000 m^−2^, making this system particularly attractive to resource-poor laboratories. We also note that bubble wrap has been used to culture cells, carry out optical trapping experiments, and the colorimetric sensing of glucose.^9–11^

In this report, we have initially chosen the synthesis of core/shell CuInS_2_/ZnS quantum dots as an example of where multiple simultaneous, parallel reactions can optimise luminescent nanoparticle synthesis; specifically, the optimum core compositions and shell precursor concentrations to achieve the brightest materials. CuInS_2_ quantum dots were chosen as they are quickly emerging as key materials for numerous applications as they do not contain heavy metals, yet exhibit excellent optical properties, notably towards the red end of the visible spectrum. Such ‘green’ quantum dots may represent the next generation of materials for use in solar and biological applications, although such ternary solid-state materials have a wide variety of phases and stoichiometries.^12^ Also, for the most basic applications, the majority of quantum dots require an inorganic shell layer to resist oxidation and confine charge carriers. Shell deposition is not simple, and often requires numerous attempts to reach the optimum thickness (bright point).^13^ If a shell is too thin, then the charge carriers are not sufficiently confined; if the shell is too thick, then the layer can exhibit defects that reduce the emission intensity. There, therefore, exists a complicated array of variables (for example, core material composition and shell thickness) that requires optimisation for core/shell quantum dot preparation.

Whilst numerous reports exist on the various synthesis methods of the Cu/In/S quantum dot system^12^ and the numerous resulting phases and materials, a key variable has not been explored in as much depth – that is, the optimum reaction conditions for luminescent core/shell materials. Determining the preferred core/shell structure in typical glassware reactions can be laborious and expensive, requiring numerous extensive, individual experiments and it would be beneficial if this could be decided simply and rapidly. In this work we report the exploration of several core precursor ratios and a shelling reaction in a few simple steps; the resulting brightest materials can be easily determined optically and then analysed and confirmed spectroscopically. We also describe how, after using bubble wrap to determine the key reaction parameters, we then carry out the entire reaction, from core synthesis to shell deposition in one single bubble, negating the need for standard glassware.

Initially, whilst referring to previous reports,^14^ we prepared core CuInS_2_ particles with a variety of Cu : In molar ratios (1 : 5, 1 : 10, 1 : 20, 1 : 40, 1 : 80), by pre-mixing the precursors for each ratio in separate vials, followed by injection of the reagents into a series of adjacent individual bubbles (*ca.* 5 mL volume, Fig. 1A for scale) on a single sheet, followed by heating the sheet in a water bath at 85 °C for 60 minutes. From these experiments, we determined by spectrometer that a range of materials with differing band edges and emissive properties resulted, confirming the variety of optical properties in this system. Materials prepared with a precursor ratio of 1 : 5 displayed absorption spectra with no excitonic features, whilst all other materials exhibited an excitonic feature at *ca.* 450 nm, although to varying degrees (ESI Fig. S1†). The emission spectra showed a similar variation, with the 1 : 5 (Cu : In) ratio materials showing no evidence of emission, whilst the 1 : 10 ratio material clearly displayed two features, at *ca*. 550 nm and 650 nm (ESI Fig. S2†). These emission features are strongly related to vacancies and defects, with the emission towards the red end of the visible spectrum reportedly associated with copper vacancies and donor–acceptor recombination.^15^ Features at *ca.* 535 nm have previously been observed by Macdonald *et al.* in CuInS_2_ prepared in water, and again attributed to defects.^16^ By increasing the Cu : In ratio, the feature at *ca.* 650 nm decreased whilst the feature at *ca.* 550 nm became predominant. Despite emission being detected by spectrometer, none of the particles visibly fluoresced in the bubble wrap under 365 nm excitation, consistent with materials which exhibit a low emission quantum yield. The absorption spectra obtained using the reported method was similar to those obtained using an earlier synthetic method which was utilised for this work; the features exhibited no excitonic features, with onsets of absorption at *ca.* 600 nm. Emission spectra for similar reactions in glassware exhibited maxima between *ca*. 550 nm and 650 nm for core/shell materials, whilst emission spectra obtained for materials prepared in bubble wrap exhibited emission between *ca.* 550 nm and *ca.* 700 nm, extended into the red by a further 50 nm.^14^

**Fig. 1:**
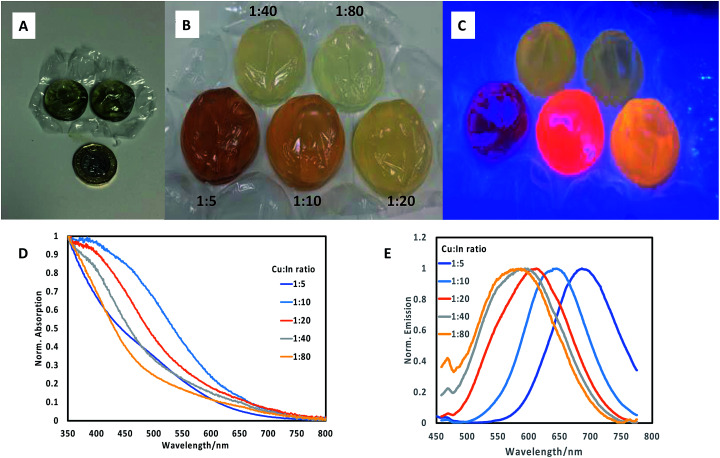
(A) Photograph of quantum dot-filled bubbles with a £1 coin for size comparison; (B) photograph of bubbles filled with CuInS2/ZnS quantum dots (prepared with differing Cu : In ratios as shown) after synthesis; (C) photograph of materials in previous figure under 365 nm excitation; (D) normalised absorption spectra of CuInS_2_/ZnS quantum dots prepared at different Cu : In ratios; (E) normalised emission spectra of CuInS_2_/ZnS quantum dots prepared at different Cu : In ratios. This figure shows the range of optical properties available by varying precursor conditions in a simple bubble wrap synthesis.

We then extended the synthesis to the preparation of a series of CuInS_2_/ZnS quantum dots in bubble wrap by repeating the core synthesis using differing Cu : In ratios as described above. Following this, a set amount of ZnS shell precursors were added to each bubble and once resealed, the sheet was then reheated to 85 °C for a further 60 minutes. The resulting array of CuInS_2_/ZnS quantum dots exhibited different colours (Fig. 1B), which were luminescent when illuminated at 365 nm (Fig. 1C), clearly emitting at different wavelengths. It was observed that the deposition of a ZnS shell on CuInS_2_ core nanoparticles prepared by different precursor ratios produced quantum dots of varying band edge positions as suggested by the difference in colour of the samples, and different emissive colours and brightness. The materials were analysed spectroscopically (Fig. 1D and E) and the range of absorption and emission wavelengths were confirmed. The absorption band edges of the core/shell materials lost the sharp profile and all excitonic features yet remained in the same approximate spectral region (normalised spectra, Fig. 1C). The most surprising results were observed in the emission spectra, where the core/shell sample with Cu : In ratio of 1 : 5 exhibited an emission profile at *ca.* 700 nm (Fig. 1E), whereas the core material alone did not display any emission. Notably, all emission appeared composed of a single feature with similar full width at half the maximum (FWHM) of *ca.* 110 nm, with no evidence of the previous secondary feature at *ca*. 550 nm, in agreement with previous studies which attributed the high energy emission suppression to zinc passivation of donor defect sites.^15,16^ Increasing the Cu : In ratio resulted in a gradual blue shift in the emission maxima, to a minimum wavelength of *ca*. 550 nm for core/shell samples prepared with a 1 : 80 core Cu : In precursor ratio. By eye, it was determined that CuInS_2_/ZnS nanoparticles with core Cu : In precursor ratios of 1 : 10 and 1 : 20 resulted in the brightest materials, although both emitted at different wavelengths. It should be stated that despite significant work into the origin of emission from Cu/In/S nanomaterials, no clear opinion has been reached; although it is clear that radiative emission does not originate from a simple quantum confined band edge; rather from an as-yet unconfirmed defect state(s).

Once we had ascertained that addition of a ZnS shell to core materials prepared with a 1 : 5 precursor ratio resulted in a luminescent structure (whereas the core material alone was non-luminescent, as determined visually and spectroscopically), we explored the potential for further enhancing the emission by varying the amount of ZnS precursors, potentially providing a thicker shell and hence exploring the opportunity to uncover a bright point. As can be seen from Fig. 2A–D, varying the amounts of shell precursor with extended heating had minimal effect on spectral position or emission brightness. Spectroscopic examination of the materials prepared in the bubbles displayed similar absorption edges (all exhibiting the same band edge position with a slight suggestion of an excitonic feature at *ca.* 500 nm as shown in Fig. 2C) whilst emission spectra appeared to reduce slightly in intensity with the addition of shell precursors (Fig. 2D) whilst maintaining the same spectral position, showing no evidence of exciton leakage into the shell (Fig. 2D, inset).

**Fig. 2:**
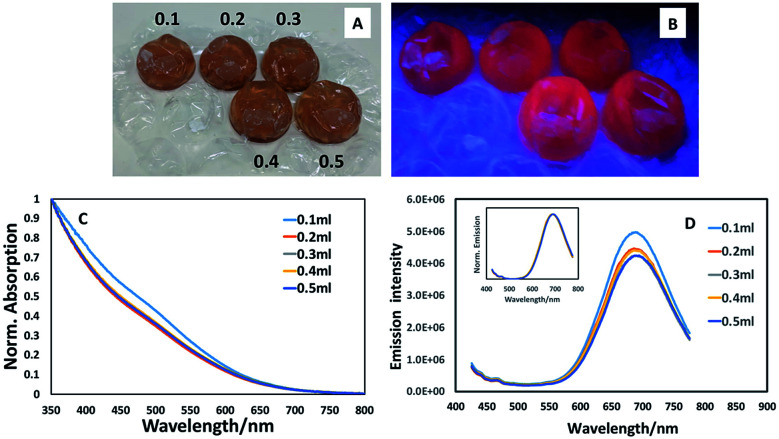
(A) Photograph of CuInS_2_ quantum dots after various volumes (numbers shown in mL) of ZnS precursor had been added; (B) photograph of materials in previous figure under 365 nm excitation; (C) normalised absorption spectra of CuInS_2_/ZnS quantum dots prepared using different amounts of ZnS precursor; (D) emission spectra of CuInS_2_/ZnS quantum dots prepared using different amounts of ZnS precursor. Inset, normalised spectra showing spectral position. The data shows that addition of varying amounts of shell precursor had little effect on the optical properties of CuInS_2_/ZnS.

To ensure that the potential of bubble wrap synthesis extends beyond simple combinatorial-style synthetic experiments (where starting materials were injected from a precursor reservoir into numerous bubbles) and to mimic typical glassware-based reaction where precursors are sequentially injected, we prepared CuInS_2_ quantum dot with a Cu : In ratio of 1 : 10, followed by addition of 0.2 mL ZnS precursor solution, entirely in a single bubble in a stand-alone experiment by sequentially injecting precursors and heating. In this case, a phosphate buffer solution of the metal precursors and capping agent (l-cysteine) were injected into a bubble then sealed. Following this, the sulfur precursor was injected and the bubble resealed, left for one hour, then heated in a water bath at 85 °C for one hour. Shell addition as described above, completed the reaction, the product of which was found to brightly luminesce when excited at 365 nm (Fig. 3A and B). Spectroscopic analysis of two identical reactions in adjacent bubbles confirmed almost identical optical band edges, whilst the emission profiles shifted position slightly (Fig. 3C and D). This confirms that pre-mixing precursors before addition to a bubble is unnecessary and that bubble wrap can be used as a stand-alone reaction vessel. Whilst the method can be easily adapted to aqueous-based quantum dot synthesis, there are obvious limitations. This method cannot, as yet, be used to make quantum dots at high temperatures in organic solvents, which is considered to be the most popular method. There are also no facilities to involve mixing, although this has not been an issue to date and we assume convective heating dominates solvent volumes as small as 5 mL, although this is clearly one factor that could impact which nanomaterial product is obtained; such issues have been previously highlighted as important in the high temperature synthesis of cobalt nanoparticles.^17^ Also, this method relies on the initial analysis of colours and intensities by eye, with no correction for mistaking optical density/colour or consideration for the eye's response to green over red, for example. One should note however, that the ultimate use for these particles may be displays, which also rely on the eye's inherent optical response. Likewise, optically-based assays have been developed that allow antigen detection at the femto-gram per milliliter level by the naked eye.^18^

**Fig. 3:**
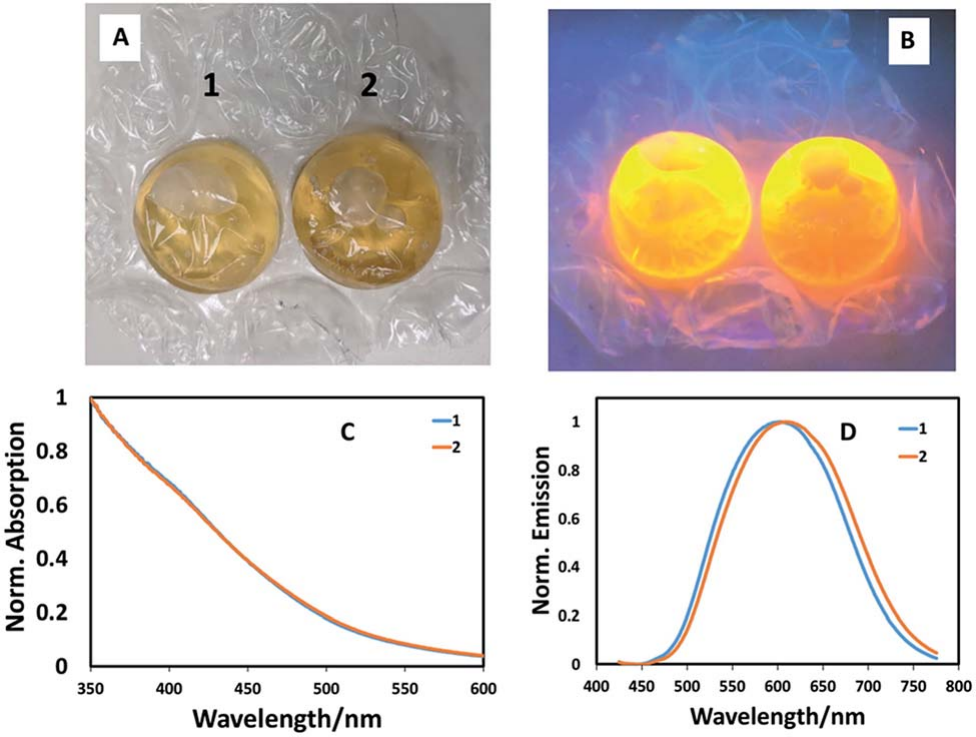
(A) Photograph of two sets of CuInS_2_/ZnS quantum dots prepared by a stand-alone method in two adjacent bubbles using the optimised conditions (Cu : In ratio of 1 : 10, followed by addition of 0.2 mL ZnS precursor solution); (B) photograph of CuInS_2_/ZnS quantum dots used in previous figure excited at 365 nm; (C) normalised absorption spectra of the two samples shown in previous figures; (D) normalised emission spectra of the two samples shown in previous figures. This data shows that CuInS_2_/ZnS quantum dots can be simply prepared using sequential precursor addition in a bubble, and that the method is reproduceable.

In conclusion, we have demonstrated that bubble wrap can be used in a combinatorial style set of experiments to determine the optimum reaction conditions for brightly luminescent core/shell quantum dots. We also demonstrated that bubble wrap can be used as a stand-alone reaction vessel, withstanding numerous reagent injections and heating, which should be applicable to other simple chemical reactions.

## Conflicts of interest

There are no conflicts to declare.

## Supplementary Material

RA-009-C9RA02018G-s001
