# Supplementary material for: A glassware-free combinatorial synthesis of green quantum dots using bubble wrap
Source: RSC Adv. 2019 May 29;9(29):16851–5. doi: 10.1039/c9ra02018g (PMC9064422; doi:10.1039/c9ra02018g)
Supplement: RA-009-C9RA02018G-s001 [file RA-009-C9RA02018G-s001.pdf]

Electronic Supplementary Material (ESI) for RSC Advances.
This journal is © The Royal Society of Chemistry 2019

A glassware-free combinatorial synthesis of green quantum dots using bubble wrap.

P. Bergstrom Mann,[a] K. Afzal,[a] N. J. Long,[b] M. Thanou,[c] M. Green.[a*]

Electronic supplementary information

Methods

Materials
Copper (II) chloride ($CuCl_2$, 97%), indium (III) chloride ($InCl_3$, 98%), thiourea (99%), L-glutathione reduced (L-GSH, ≥99.0%), L-cysteine (97%), sodium sulfide nonahydrate ($Na_2S.9H_2O$, >99.99% trace metal basis) and phosphate buffered saline (PBS, tablets) were obtained from Sigma Aldrich. Zinc acetate dihydrate ($Zn(OAc)_2.2H_2O$, analytical reagent grade) and 2-propanol (IPA, HPLC grade) were supplied by Fisher Chemicals. Clear nail varnish (Rimmel) was purchased from Superdrug (London, UK). Small and large bubble wrap (Aircap Handi Roll) was obtained from Sealed Air. Sterican Luer lock needles (27G) were purchased from Yorlab. Ultrapure water (Direct-Q3 system, Millipore) was used throughout and materials used as received unless otherwise stated.

Stock Solutions

PBS solutions were prepared by dissolving one PBS tablet in 200 mL of water.

Stock Solution	Component(s)	Mass (mg)	No. of mmoles	Volume of solvent[a]	Concentration (mol dm^{-3})	Storage
Cu^{2+}	$CuCl_2$	3.4	0.02	20	0.001	Ambient
In^{3+}	$InCl_3$	1106	5	5 (EtOH)	1	Ambient
L-cysteine	L-cysteine	2459	8	20 (PBS)	0.4	4°C
Na_2S	$Na_2S.9H_2O$	4804	20	20	1	4°C
ZnS Precursors[b]	$Zn(OAc)_2.2H_2O$	176	0.8	20	0.04	4°C
	Thiourea	61	0.8		0.04	
	L-GSH	368	1.2		0.06	

Synthesis

Combinatorial Synthesis of Quantum Dots in Bubble Wrap.
In a typical synthesis of Cu-In-S QDs (Cu:In, 1:40), 1 ml of Cu^{2+} solution (0.001 mmol), 0.04 ml of In^{3+} solution (0.040 mmol) and 6.1 mg of L-GSH (0.02 mmol) (or 0.05 ml of L-cysteine solution) were added to a three-neck round bottom flask and diluted with 20 ml of PBS. The volumes of Cu^{2+} and In^{3+} solutions required for other ratios are included in the table below. Under vigorous stirring, 0.062 ml of Na_2S solution was injected rapidly and the solution immediately turned from colourless to yellow. Aliquots of QD solution (4 mL) were injected into large bubbles (2.5 cm across) through a 27-gauge needle. It is estimated that each bubble had a volume of ca. 5 mL. Care was taken not to leave a significant air bubble in the compartment. The resulting hole was sealed with a liberal application of nail varnish and left to dry for up to 1 hour. Sealed bubbles were submerged and heated in a water bath at 85°C for 1 hour.

Cu:In Ratio	Vol. of 0.001 M Cu solution (mL)	Vol. of 1 M In solution (µL)
1:5	1.0	5
1:10	1.0	10
1:20	1.0	20
1:40	1.0	40
1:80	1.0	80

Direct Synthesis of Quantum Dots in Bubble Wrap.
A solution of $CuCl_2$ (0.25 µmol), $InCl_3$ (10 µmol) and L-cysteine (160 µmol) in PBS (4 mL) was prepared, injected into a single large bubble and the bubble sealed. Subsequently, Na_2S (62 µmol) was injected rapidly with a 27-gauge needle and the hole sealed with nail varnish as above. After leaving to dry from 10 to 60 minutes, the bubble was heated in a water bath at 85°C for 1 hour.
ZnS Shelling Procedure. To a typical bubble containing 4 mL of QD solution, ZnS precursor (0.2 mL) was added with a 27-gauge needle, the hole sealed with nail varnish and left to dry for 1 hour. Sealed bubbles were submerged and heated in a water bath at 85°C for a further 1 hour.

Characterisation

Absorption spectroscopy measurements were recorded using a Hitachi U-4100 UV-visible-NIR spectrophotometer in a quartz cuvette (pathlength of 1 cm). Photoluminescence spectra were recorded on a Fluoromax 4 (Horiba) spectrofluorometer with samples in quartz cuvettes (pathlength of 1 cm).

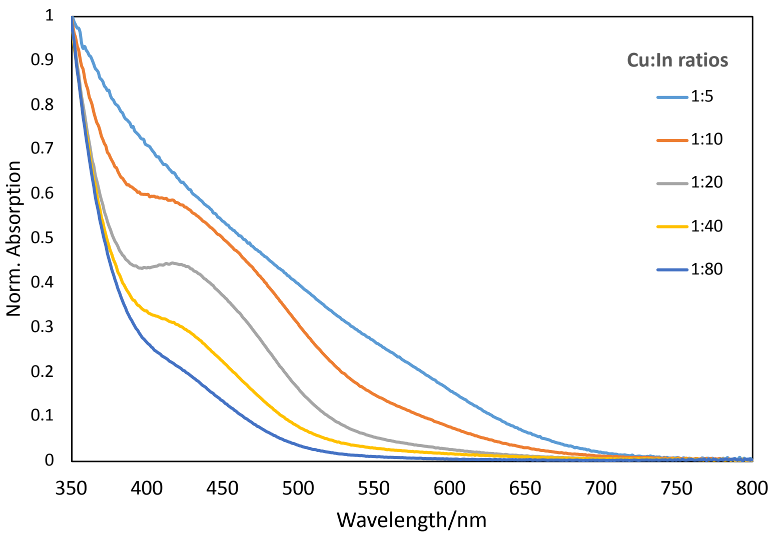

Supporting figure 1 – absorption spectra of $CuInS_2$ particles prepared using different Cu:In precursor ratios. (In bubble wrap, 85 °C, 60 mins heating).

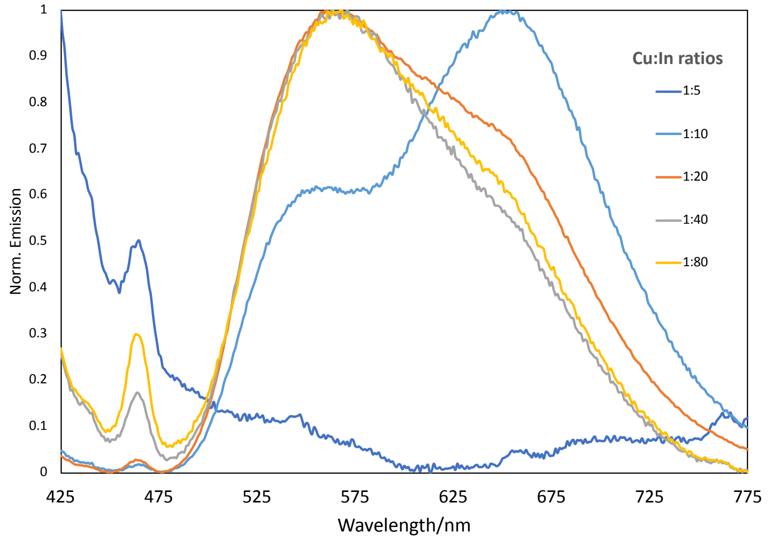

Supporting figure 2 – Emission spectra of $CuInS_2$ particles prepared using different Cu:In precursor ratios. (In bubble wrap, 85 °C, 60 mins heating)
